# EchoGrid: High-Throughput Acoustic Trapping for Enrichment of Environmental Microplastics

**DOI:** 10.1021/acs.analchem.4c00933

**Published:** 2024-05-25

**Authors:** Martim Costa, Björn Hammarström, Liselotte van der Geer, Selim Tanriverdi, Haakan N. Joensson, Martin Wiklund, Aman Russom

**Affiliations:** † KTH Royal Institute of Technology, Division of Nanobiotechnology, Department of Protein Science, Science for Life Laboratory, 171 65 Solna, Sweden; ‡ KTH Royal Institute of Technology, Department of Applied Physics, Science for Life Laboratory, 171 65 Solna, Sweden; § AIMES − Center for the Advancement of Integrated Medical and Engineering Sciences at Karolinska Institutet and KTH Royal Institute of Technology, 114 28 Stockholm, Sweden

## Abstract

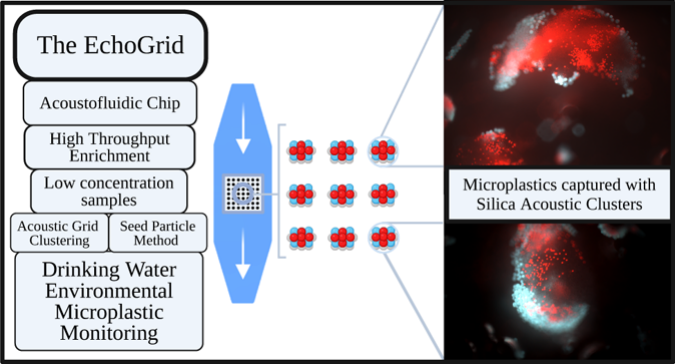

The health hazards of micro- and nanoplastic contaminants in drinking water has recently emerged as an area of concern to policy makers and industry. Plastic contaminants range in size from micro- (5 mm to 1 μm) to nanoplastics (<1 μm). Microfluidics provides many tools for particle manipulation at the microscale, particularly in diagnostics and biomedicine, but has in general a limited capacity to process large volumes. Drinking water and environmental samples with low-level contamination of microplastics require processing of deciliter to liter sample volumes to achieve statistically relevant particle counts. Here, we introduce the EchoGrid, an acoustofluidics device for high throughput continuous flow particle enrichment into a robust array of particle clusters. The EchoGrid takes advantage of highly efficient particle capture through the integration of a micropatterned transducer for surface displacement-based acoustic trapping in a glass and polymer microchannel. Silica seed particles were used as anchor particles to improve capture performance at low particle concentrations and high flow rates. The device was able to maintain the silica grids at a flow rate of 50 mL/min. In terms of enrichment, the device is able to double the final pellet’s microplastic concentration every 78 s for 23 μm particles and every 51 s for 10 μm particles at a flow rate of 5 mL/min. In conclusion, we demonstrate the usefulness of the EchoGrid by capturing microplastics in challenging conditions, such as large sample volumes with low microparticle concentrations, without sacrificing the potential of integration with downstream analysis for environmental monitoring.

## Introduction

The modern world has witnessed an exponential increase in plastic production, usage, and waste over the past 70 years as industries develop greater demand for these materials. In 2023 alone, over 390 million metric tons of plastics were produced globally,^1^ and this is reflected in its ubiquitousness throughout society. The use of plastics is justified by its advantages as a cost-effective and versatile material for a myriad of applications that benefit from rapid prototyping, disposability, and resistance to degradation.^2^ While the use of plastics has brought substantial commercial and industrial benefits, it also poses a significant risk to both the environment and public health. Plastic contaminants of different types and sizes have been discovered in ecosystems ranging from the arctic to tropical ocean surface water,^2−4^ highlighting the long reach of their durable contamination throughout the world.

The widespread contamination of microplastics has raised concerns about the hazards that these pollutants may pose to the environment and human health. This concern is growing as evidence of microplastics pollutants in everyday consumer products and drinking water^5^ continues to increase. Plastic pollutants are known to be very heterogeneous,^6^ which poses a significant challenge of developing systems capable of addressing the need for systematic and reliable monitoring.^7,8^ Microplastics appear in various forms, such as fibrous, rounded, or irregular shaped fragments, and can be composed of different materials such as polystyrene, polyethylene, and rubber.^9,10^ The process by which these contaminants leach from larger bodies of plastics and disperse throughout their environment is complex, and much is still unknown. While there are approaches to investigate how these polymers move through the environment,^11,12^ it remains a challenge to create a system that is capable of enriching these analytes from high volumes of sample in a way that can be integrated with modern analytical techniques.^13^ This is an essential step for improved categorization, monitoring, and ultimately management of this environmental and health hazard.

Microfluidics has been widely used to accurately manipulate micro- and nanoparticles in miniaturized fluidic channels. In this multidisciplinary field, fluidic devices are widely applied in the biomedical field for applications such as DNA amplification,^14^ feed screening,^15^ and particle separation^16^ and enrichment.^17^ Among these, particle enrichment is of particular interest to environmental and industrial monitoring due to the low concentrations often found in environmental and food samples; when compared to biological samples, which are collected at micro- to milliliter volumes with comparatively higher concentrations of analytes, environmental contaminants are found at lower concentrations and in larger volumes. For this reason, technologies that are capable of processing larger volumes of fluid while enriching these small particles are highly relevant.

Acoustofluidics combines microfluidic structures with acoustic actuation.^18^ This combination of technologies enables the manipulation of microscale objects within a microchannel, without direct contact, through the use of acoustic forces. Acoustofluidics has been used for a wide range of biomedical applications, such as the separation of bacteria from blood cells,^19^ the purification of microvesicles,^20^ and separation of cells from complex mixtures.^21^ For environmental applications, acoustofluidics has been utilized for in-flow imaging of microalgae^22^ and flow through focusing of microplastic particles.^23^ However, effective enrichment of microparticles at throughputs relevant for environmental monitoring applications remains a challenge. Acoustic trapping is particularly relevant in this context as it is capable of completely arresting particles in flow to provide very high enrichment factors while interfacing well with downstream analysis as demonstrated in extracellular vesicle enrichment^24^ or bacterial analysis.^25^ Continuous development in this area has focused on increasing flow rates, increasing capture efficiency, decreasing the minimum particle size, automation, and also predictable particle patterning. Strategies to fulfill these objectives have emerged as new techniques, such as the seed particle method,^26^ acoustic tweezers,^27^ and using actuation methods based on bulk acoustic waves (BAWs),^28^ surface acoustic waves (SAWs),^29^ and traveling surface acoustic waves (TSAWs).^30^ Other acoustic phenomena, such as acoustic streaming,^31^ have also been used as a solution to focus submicrometer particles, as these are harder to manipulate using direct acoustic radiation forces. To the best of our knowledge, the seed particle method has not been used for microplastic enrichment applications. However, seeded acoustic traps for nanoparticles are known to usually operate below 100 μL/min,^25^ with higher flow rates such as 2 mL/min coming at the cost of more than 90% of particles.^24^ In order to extract a relevant microplastic sample from a large volume for enrichment and detection, it is necessary to have a device that is compatible with high flow rates while being capable of particle capture at a statistically relevant efficiency.

To address the challenge of enriching microplastics from dilute samples at high throughput toward analysis, a surface displacement transducer (SDT) was used as a single-axis actuator capable of delivering patterned acoustic trapping in a microchannel. We show how the combination of this highly energy-efficient transducer with a microfluidic channel system defined in PDMS offers a highly reproducible, flexible, low-cost, and easy to fabricate system with high performance. The SDT-based trapping platform, termed the EchoGrid, is capable of enriching microplastics of various sizes from flowing suspensions at flow rates exceeding 5 mL/min. The 3D acoustic spatial control offered allows the arrangement of the captured plastic contaminants in defined geometric arrays, a potential advantage for end point analysis based on microscopy or spectroscopy. In addition, the seed particle method is applied by using silica particles (which are inert for detection methods such as Raman spectroscopy) as a way to enrich lower-concentration samples of large volumes. In previous works the seed particle method has primarily been used as a method to capture nanoparticles,^26^ but in this work we demonstrate how major advantages are gained also when addressing larger particles at low concentrations where high flow rates and concentration-independent performance is critical. Preloading the acoustic trap with silica seed particles addresses a limitation in acoustic trapping, which is the comparatively poor capture efficiency for initial single particles before larger clusters have been formed when the sample particles are present at low concentration, and high throughput is needed. For this reason, we study and compare clustering and enrichment performances between unloaded and preloaded traps.

## Materials and Methods

### Acoustic Trapping Device

#### Fabrication

**Figure 1:**
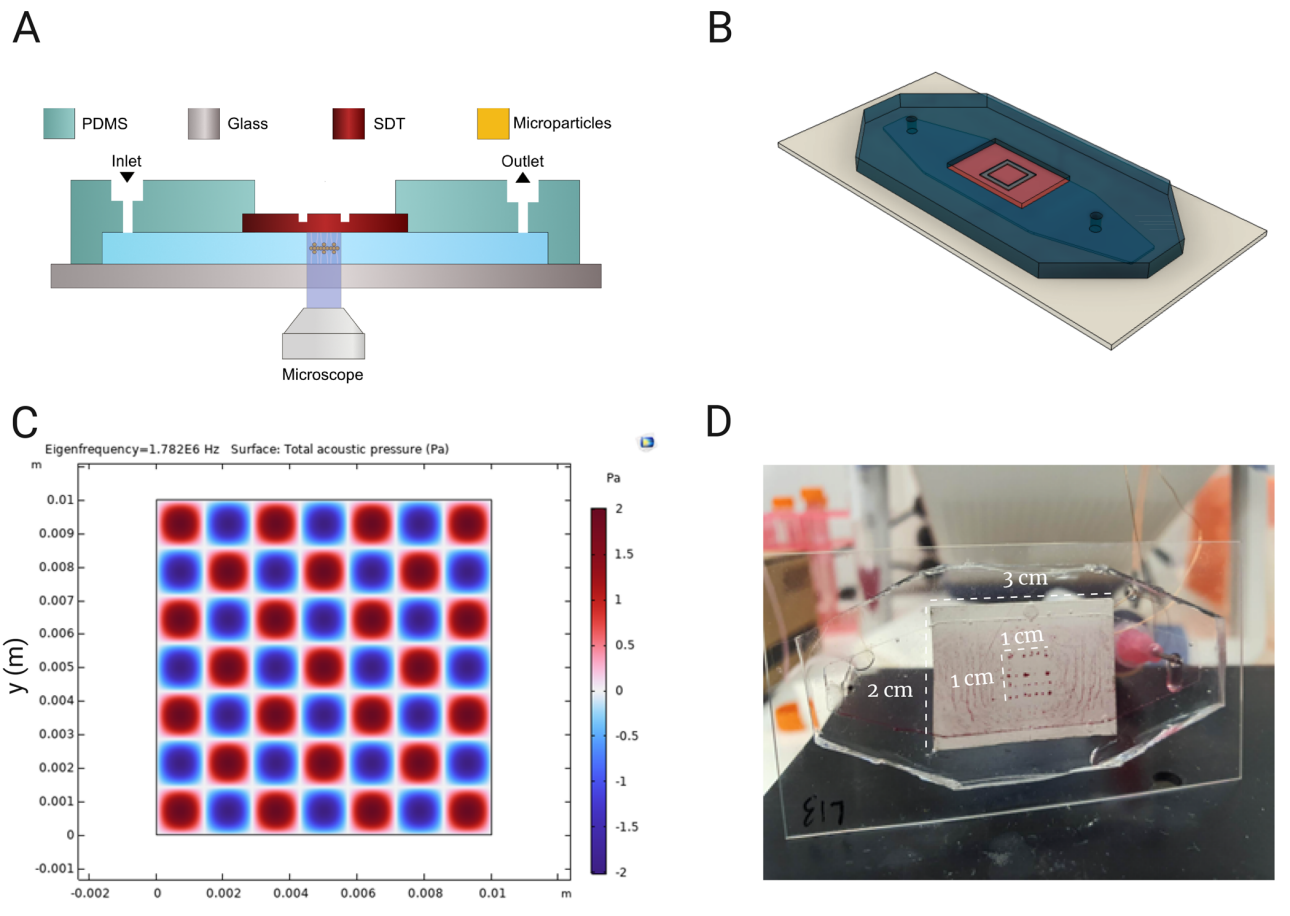
(A) Schematic side-view of the EchoGrid device and (B) a 3D rendering of the complete assembly. (C) A 2D simulation of the lateral resonances in the 10 × 10 mm^2^ piezoelectric protrusion at one of its eigenfrequencies, exhibiting a 7 by 7 grid. (D) EchoGrid device during testing. The active area of the SDT can be seen patterned with red-colored 10 μm particles in a 7 by 7 grid at 1.782 MHz.

The device consisted of three main parts: a micromachined piezoelectric transducer, a polydimethylsiloxane (PDMS) layer with microchannels, and a glass reflector layer. The device is shown schematically in Figure 1A,B and during operation in Figure 1D. The piezoelectric transducer (Pz-26, Parker Meggitt, UK) had a dimension of 30 × 20 mm^2^. The wrap-gating connected the top electrode to the back of the piezoelectric plate, such that both electrical connections could be soldered to the back of the plate. The design of the trapping area was done using AutoCAD and Fusion 360 (Autodesk Inc., CA, US) to generate the milling files. A 2 mm diamond-tipped endmill was used in a computerized numerical control (CNC) milling machine (Modela MDX-40A 3D Milling Machine, Roland DGA, CA, US) with cutting parameters optimized for precise height and border control. A 0.1 mm-deep indent was milled into the backside of the piezoelectric transducer. This resulted in an SDT where the residual protrusion defined the trapping region,^32^ as shown in Figure 1A.

The polydimethylsiloxane (PDMS) microchannel was cast using a poly(methyl methacrylate) (PMMA) mold. This mold was milled from a 0.5 mm PMMA substrate and then attached to a molding surface. A digital micrometer (293 QuantuMike, Mitutoyo, JP) was used to verify the precision of milled channel height (390 μm). Utilizing standard soft lithography techniques, PDMS was mixed in a 10:1 ratio of monomer to curing agent (SYLGARD 184, Dow Corning, MC, US). The micromachined transducer was placed in the mold, and PDMS was cast around it, degassed for 45 min, and cured at 60 °C for 90 min. Subsequently, the PDMS layer with the integrated transducer was extracted from the mold and pierced for the fluidic connections with a 20 ga syringe (Instech Laboratories Inc., PA, US). The glass reflector layer was a 1 mm thick borosilicate double-width microscope slide (Corning Inc., NY, US) setting the total footprint of the device to 75 × 38 mm^2^, with an internal channel volume of 309 mm^3^. As a final step in the fabrication process, the PDMS chip with the integrated transducer was bonded to the glass reflector layer by using an oxygen plasma surface treatment.

#### Modeling

Two theoretical models were used to predict the behavior of the device. Initially an analytical 1D model was used to determine the main axial resonances given by the material layers composing the transducer, fluid channel, and the reflector. This model was based on an acoustic transmission line^33^ and is described in the device section. Subsequently, a 2D model of the lateral resonances was implemented in COMSOL Multiphysics (COMSOL 6.1, COMSOL Multiphysics, Sweden) to find suitable lateral dimensions of the SDT-protrusion. Here, the shear velocity of the piezoelectric material, 3600 m/s, was used in an eigenmode simulation to find the resonance patterns defining trapping in the fluid cavity, as shown in Figure 1C. Every device was subjected to an impedance sweep (Z-Check 16777k, Analog Instruments) to evaluate the correct positioning of the admittance peaks. In a piezoelectric transducer the peaks in the admittance represents mechanical resonances. For this device, two admittance peaks were expected to occur: one at 1.750 MHz and one at 2.100 MHz, around which well-defined and robust acoustic trapping in a grid was expected to occur. Before usage, each prototype was filled with a high concentration of red 10 μm polystyrene particles (61946, Sigma-Aldrich, Switzerland) and actuated according to simulation predictions, at 8 V_pp_. From these experiments, two frequencies stood out in forming a grid with well-defined clusters, 1.782 and 2.020 MHz.

## Experimental Section

### Setup and Samples

The experimental setup consisted of the following equipment: a signal generator (DS345, Stanford Research Systems, CA, US), an oscilloscope (PicoScope 3205, Pico Technology, UK), a 4× current amplifier (ADA4870ARR-EBZ, Analog Devices, MA, US), a syringe pump (PHD Ultra, Harvard Apparatus, MA, US), and a fluorescence microscope (Axiovert 135M, Carl Zeiss AG, Germany). The fluorescence images used for evaluating particle capture were all captured with an exposure time of 80 ms, a gain of 30, and at 1× magnification. Plastic tubing (BTPE-60, Instech Laboratories Inc., PA, US) with metallic tubing connectors (SC20/15, Prime Bioscience, Singapore) was used to establish fluidic connections to plastic syringes (Plastipak, BD Bioscience, NJ, US).

Particle suspensions were created by adding polystyrene particles to deionized water supplemented with 1 wt % detergent (Tween-20, Sigma-Aldrich, Switzerland). Working solutions of 10^6^ and 10^4^ particles/mL were confirmed by using an automated cell counter (Countess 3 FL, Invitrogen, MA, US). The polystyrene particles used were: 2 μm (Red Fluorescent Fluoro-Max, 542/612 nm, ThermoFisher, CA, US), 10 μm (Red Fluorescent FluoSpheres, 580/605 nm, Invitrogen, MA, US), and 23 μm (Green Fluorescent Fluoro-Max, 468/508 nm, ThermoFisher, CA, US). The 10 and 23 μm particles are shown as yellow and blue, respectively, in their separate experiments. In the experiments where they are mixed, the 10 μm particles are depicted as red and the 23 μm particles as blue. For seed particle experiments 10 μm silica particles (Sigma-Aldrich, Switzerland) were used at a concentration of 10^8^ particles/mL. Deionized water was used to prime the microchip, and 50% ethanol was used to clean the chip between experiments.

### Experimental Procedure

Two methods for enrichment of microplastics were evaluated: direct capture, as illustrated in Figure S1A, and seed-particle-assisted capture, as shown in Figure S1B. Throughout these experiments, a range of flow rates (20 μL/min to 50 mL/min) and particle sizes (2, 10, 23 μm) was evaluated.

In the direct capture method, a sample containing microparticles flowed through the chip with the acoustic field turned on. By these means microparticles were enriched through acoustic trapping above the SDT. At the starting time point, *t* = 0, an initial fluid sample was collected at the outlet and a fluorescence image was captured over the trap. Collection of particle samples and fluorescent images was then repeated with regular intervals throughout the experiment in accordance with the selected flow rate. The particle capture efficiency was calculated using the formula (1 – (particle concentration at the inlet/particle concentration from the outlet)) × 100.

In the silica-enhanced seed-particle method, the acoustic trap was preloaded with silica particles before flowing the sample through the chip. At first, the channel was filled with a suspension containing silica beads at high concentration (10^8^ particles/mL) to ensure complete and uniform coverage of the active trapping area. Subsequently, the acoustic field was turned to create a silica grid. The excess silica was then washed out from everywhere except the acoustic nodes at a flow rate of 15 mL/min. Finally, the sample containing microplastics was loaded, and capture occurred in the silica clusters. At every time point, a fluid sample was collected from the outlet, and a microscope image of the grid was acquired.

The sampling intervals were adjusted to match the experimental flow rate and consider the maximum possible volume of the syringe (20 mL). Particle concentrations in the fluid samples were established using a manual hemacytometer; an automated cell counter; or, in the case of the silica experiments, an ImageJ cell counting plugin. In the analysis of the fluorescence images, the image region just before the trap was subtracted from the fluorescence over the trap to obtain normalized intensities within each image. The image sequence was subsequently normalized to evaluate microplastic enrichment over time.

## Results and Discussion

### Acoustofluidic Device

The developed microfluidic device is an assembly of three main components: a 2 MHz piezoelectric transducer, a glass reflector, and a PDMS structure with microchannels. This model follows previous work^32^ based on Glynne-Jones^33^ and Krimholtz,^34^ where a highly efficient resonator can be constructed according to the Krimholtz, Leedon, and Matthae (KLM) model.^34^ This model allows for the determination of the fundamental resonances of one-dimensional multilayer structures. The KLM model is also useful when designing 3D systems such as the device used in this study.

In this work, a microfluidic module was assembled that integrated a piezoelectric transducer within a PDMS microchannel, creating a half-wavelength layered resonator (Figure 1A). The microfluidic design takes advantage of the deformability of the PDMS to grant structural integrity to the microchannel while allowing for direct contact of the SDT with the liquid, maximizing the device’s efficiency. The setup allows for a powerful axial trapping force, forcing particles to be at the center of the channel. However, this force is not sufficient to ensure particle trapping in the direction along the length of the microfluidic channel as the flow rate is introduced. By micromilling the back of the transducer (Figure 1B), it is possible to select a trapping area on the opposite side of it. The dimensions of this cut are calculated in such a way that a lateral resonance is created within the microchannel, generating a trapping pattern that traps and retains particles against fluid flow. With the combination of these two effects, particles can be clustered axially, in the levitating direction, and laterally along the channel direction according to the acoustic potential of the active surface. The spatial performance of this type of transducer, termed a surface displacement transducer (SDT) from now on, can be simulated. In Figure 1C, the acoustic pressure surface simulation using COMSOL Multiphysics is presented, and the corresponding experimental counterpart is shown in Figure 1D. The height of the microfluidic channel and the lateral dimension of the milled slot in the piezo, which ensures matching with the lateral resonance of the device, are important parameters that determine the trapping power of the device. The lateral resonance is the major force responsible for counteracting the Stokes’ drag of the flowing particles and maintaining them in the clusters that compose the grid. The grid can be tuned in real time by switching the frequency with the signal generator. The agreement between simulation and experiment indicates that the fabrication methodology of the device is robust, considering that any deviation in the milled dimensions of the SDT or in the height of mold would result in a loss of trapping power in the resonant cavity.^35^

### Characterization of the Echogrid

#### Particle Behavior in the Acoustic Field

A qualitative study of EchoGrid was performed to understand how different particles are affected by the acoustic field. An eigenfrequency that can achieve an organized trapping pattern was selected to investigate the capture performance of this device. The adequate transducer dimensions were calculated using COMSOL Multiphysics, sweeping eigenfrequencies to provide an overview of the various possible cluster conformations. Afterward, an in-house MATLAB script was used to determine the ideal channel height for maximum trapping power considering the piezo thickness and resonant frequency. The device was constructed according to these specifications, characterized, and experiments were conducted at low flow rates to determine the smallest particle size that is possible to pattern. Experimentally, we were able to capture 2 μm particles into a pattern (Figure S2A). As shown in Figure S2A–C, we observed three different modes of operation around one of the impedance peaks of 1.780 MHz. The other equivalent peak was 2.020 MHz.

There are two competing acoustic phenomena in the microchannel: acoustic trapping and acoustic streaming. The acoustic energy inside the system must be tuned to ensure orderly trapping; otherwise, small changes in frequency can result in excessive acoustic streaming. This prevents particles from being trapped in a grid. Small microparticles (2 μm), unlike larger ones, are more susceptible to fluid forces emerging from acoustic streaming rather than the direct trapping by the primary radiation forces (*F*_PRF_) of the acoustic waves themselves, which scale with *r*^3^. This characteristic allows the selection of the frequency that minimizes the acoustic energy that goes into streaming and maximizes that which is used for trapping, as any acoustic streaming becomes clearly visible (Figure S2C). Once trapped, the clusters are held together by the secondary acoustic radiation forces, a particle–particle phenomenon that pulls them toward one another.^26^

After selecting the frequency, we evaluated how the trapping performance of the setup is affected by several factors, namely, particle size, flow rate, and particle concentration.

#### Enrichment of Microplastics

**Figure 2:**
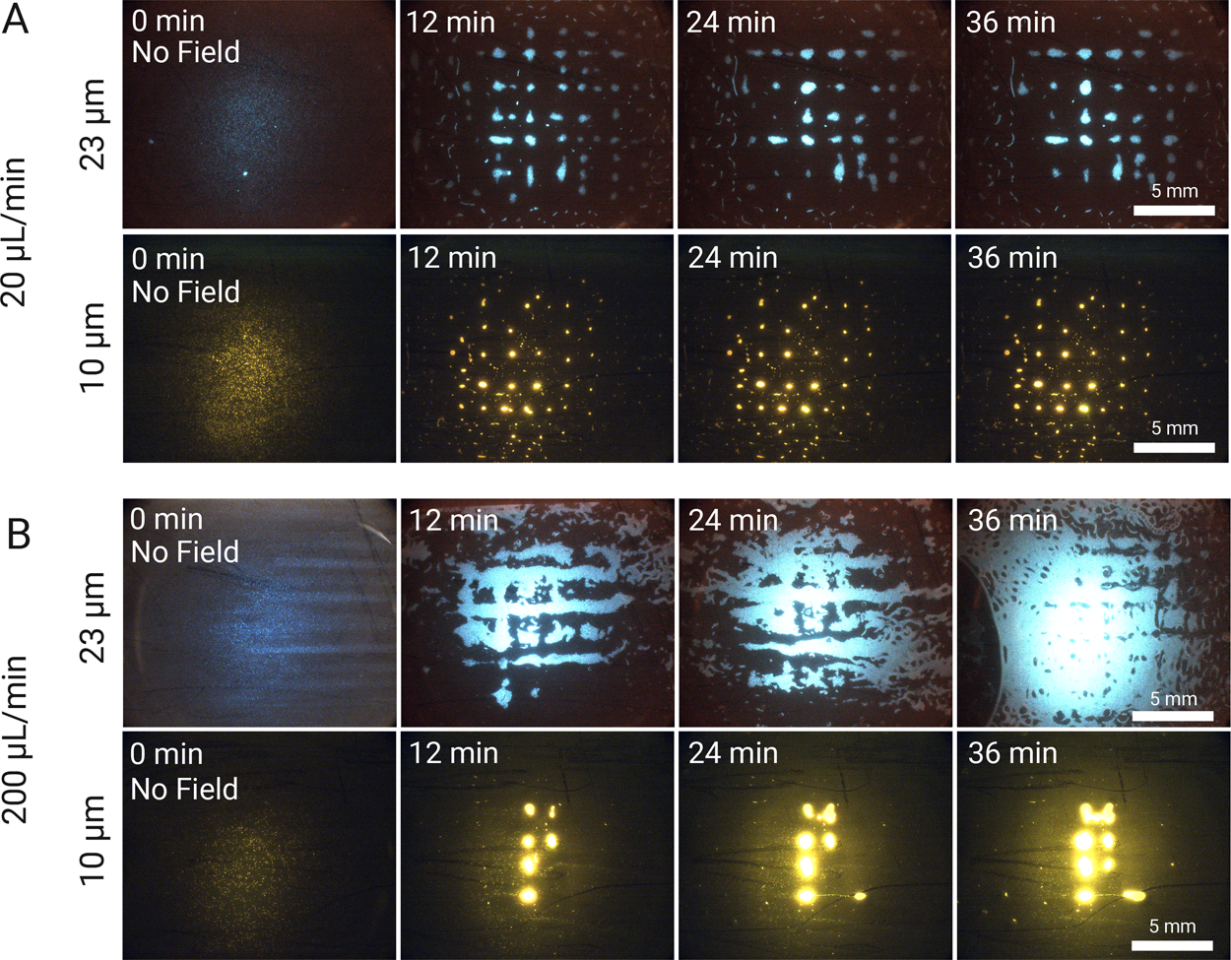
EchoGrid performance at a concentration of 1 × 10^6^ particles/mL. (A) Fluorescence image sequence for 23 (blue) and 10 μm particles (yellow) at 20 μL/min. (B) Fluorescence image sequence for the 23 and 10 μm particles at 200 μL/min. Actuation parameters: 11.60 V_pp_ for 23 μm particles and 19.20 V_pp_ for 10 μm particles, both at a frequency of 2.020 MHz. The fluid flows from left (inlet) to right (outlet), and all experiments were done in triplicate.

Two particle sizes (10 and 23 μm) were used to evaluate the trapping performance. The results are shown in Figure 2. At high particle concentrations (10^6^ particles/mL), we observe a higher capture rate after a minimum cluster size is achieved. In the absence of clusters, the probability that particles will hit an acoustic node directly and become trapped is diminished. Conversely, as clusters form and expand, more particles become close enough to be attracted and trapped by *F*_SRF_, much like a nucleation phenomenon. This highlights the synergetic effect of *F*_PRF_ and *F*_SRF_ in experimental conditions with high particle concentrations.

With regard to the flow rates, in the 20 μL/min experiment, nodes on the left side of the grid (from where the fluid flows in) are bigger than those toward the outlet. However, in the 200 μL/min case (Figure 2B) we can observe a different situation. Due to the greater flow rate, the particles require a stronger acoustic force to be captured. For the case of 23 μm particles, we note that more particles are captured by the grid to the point where the trap quickly becomes overloaded with particles. As this happens, the nodes lose their well-defined boundaries and instead interconnect with lines of particles forming between them. By the end of the experiment, the entire trapping area is filled with trapped 23 μm particles that can even resist perturbation from a bubble flowing in (see Figure 2B, 23 μm at *t* = 36 min). This resilience of the acoustic field to bubbles by the SDT was also reported by previous work,^32^ albeit outside of a continuous flow regime.

For the 10 μm particles at 200 μL/min, only the central clusters are visible and are expanded through enrichment. This is due to the nucleation phenomenon mentioned previously. In the central nodes of the active area, the trapping force is stronger,^32^ which forms the initial clusters more easily. Once these are formed, the microplastics themselves act as seed particles, aiding the more complicated task of capturing the smaller 10 μm particles, as they are less susceptible to *F*_PRF_, and improving enrichment outcomes.

We also tested low particle concentration (10^4^ particles/mL) and observed that the system does not have the same enrichment capability at low flow rates and low concentrations (see Figure S3). At 20 μL/min we observe the formation of a scarcely detectable grid that portrays difficulty in growing due to the low probability of individual microparticles flowing into the center of the nodes. At 200 μL/min, the grid is more visible. Larger flow rates carry a larger number of particles per unit of time, increasing the probability of cluster formation. In the case of 10 μm particles, we observe that there is almost no capture above the background. Based on these supporting experiments, a further increase in the flow rate would not result in particle capture at low concentrations. It is for this reason that it was relevant to develop another method beyond direct capture compatible with reducing particle concentrations while increasing throughput.

**Figure 3:**
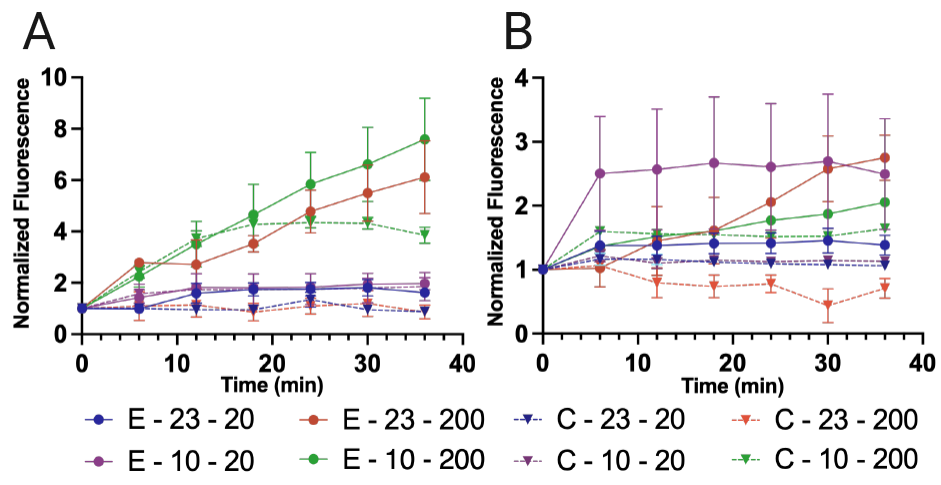
(A) Normalized fluorescence vs time at a concentration of 10^6^ particles/mL of 10 and 23 μm particles at a flow rate of 20 and 200 μL/min. (B) Normalized fluorescence vs time at a concentration of 10^4^ particles/mL of 10 and 23 μm particles at a flow rate of 20 μL/min and 200 μL/min. Experiments with acoustics field [E] are depicted with full lines and controls, no field, and [C] with dashed lines. The labels are identified by Type (E or C): particle size (μm) – Flow Rate (μL/min). The average values (*n* = 3) are shown with the standard error of the mean as error bars.

By comparing the initial flow section of the device with the central acoustic enrichment area, it is possible to extract a trend of fluorescence increase throughout the experimental time. To better illustrate the enrichment profile of the system, all fluorescence readings were normalized to the initial point of the experiment before the acoustic field was actuated, while the solution was already flowing. The average fluorescence signal of the cluster grids is shown in Figure 3.

At a high particle concentration (10^6^ particles/mL, see Figure 3A), the higher flow rate (200 μL/min) results in faster particle enrichment for both particle sizes. The particle clusters increase in intensity throughout the 36 min of the experiment and have not reached saturation. Note that the C-10-200 experiments also have an increasing signal until the 18 min mark before stabilizing. The highly fluorescent 10 μm particles flow into the channel and interact with its surface in the absence of an acoustic field, artificially increasing fluorescence signal through nonspecific binding aided by sedimentation, until the steady state between particles that enter and exit is achieved. For the lower flow rate (20 μL/min), low fluorescence intensity comparable to those from the control experiments are observed. The reason for the difference with the higher flow rate is because there are not enough particles to create clusters that are big enough to attract particles flowing through the microfluidic channel. This is also why we do not observe significant fluorescence increase over time. At the lower particle concentration (10^4^ particles/mL, see Figure 3B), both particles flowing at 200 μL/min resulted in slowly increasing fluorescence signal over time. This effect is more notable for the 23 μm particles because of particle sedimentation as there is no acoustic field to levitate the particles.

We also collected the sample at the outlet, and the corresponding particle counts are shown in Figure S4. To quantify the particle capture efficiency, the sample that remained inside the channel was calculated as a percentage of the inlet concentration. This is done by subtracting the particles counted at the outlet by those inserted at the inlet. At 20 μL/min with a concentration 10^6^ particles/mL, for the 23 μm case, we observe a capture efficiency of 98.7% at 36 min. For 10 μm, the capture efficiency reaches 81.5% at 36 min. For the 200 μL/min flow rates, for 23 and 10 μm, respectively, we observed capture efficiencies of 88.3% and 46.8% by the end of the experiment. As evidenced by these results, there is a trade-off between capture efficiency and throughput for the high-concentration particles. For the lower concentration (10^4^ particles/mL), we could not observe a clear trend (see Figure S4B). This is because, at low concentration (10^4^ particles/mL), we do not have enough particles to create clusters that are big enough to capture new particles through the *F*_SRF_—there is no nucleation possible. One way to address the challenge of low particle concentration is to preseed particles upfront, and this is described in the following section.

### Silica Preseeding for Ultrahigh-Throughput Microplastic Enrichment

#### Retaining Preseeded Silica Clusters

The particle seed method has been used mostly for nanoparticle capture in acoustofluidics since the first work that describes it.^26^ Here, we exploit the particle seeding method for low-concentration solutions of microplastics having sizes similar to those of the seed particles.

While polystyrene particles have generally been used as seed particles, silica particles are an attractive alternative.^20^ Their distinct chemical fingerprint makes them attractive for end point analysis as well as their increased trapping performance compared to polystyrene and a distinct chemical fingerprint. This is primarily because of the greater density of silica particles (2 kg/m^3^) compared to polystyrene particles (1 kg/m^3^), although other material properties such as compressibility also have a significant impact.

**Figure 4:**
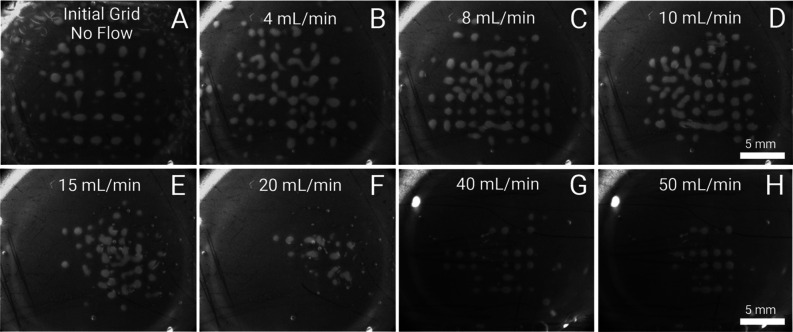
Silica grid state under increasing flow rate conditions at 14.40 V_pp_ and a frequency of 2.020 MHz. All silica particles used were 10 μm. (A) Original grid without flow, and at a flow rate of (B) 4 mL/min; (C) 8 mL/min; (D) 10 mL/min; (E) 15 mL/min; and (F) 20 mL/min. (G and H) Experiment at 26.80 V_pp_, with a flow rate of (G) 40 mL/min and (H) 50 mL/min.

As a first step, we investigated how silica particles would behave in the acoustic field and the highest flow rate that could be achieved before the clusters were displaced. A schematic representation of the methodology can be seen in Figure S1B. As seen in Figure 4, the silica clusters can withstand at least a flow of 50 mL/min with an intact grid. The fluid velocities at different flow rates tested and their associated Reynolds numbers (*Re*) can be found in Table S1. A major challenge for a stable acoustic field is temperature regulation, as heating changes the acoustic properties of the medium. In our system, the high flow rates also provide a cooling effect that counteracts the heating of the SDT as it operates at higher power. With more optimization, flow rates greater than 50 mL/min can be achieved, although it becomes challenging to use conventional microfluidic syringe pumps to handle large volumes.

In the field of acoustofluidics, to our knowledge, a grid enduring at 50 mL/min is a considerable improvement in the state of the art, even from the perspective of fluid velocity.^24,36^ The extremely high flow rates (*Re* ≈ 100) achieved in this work are not common in microfluidics-based separation methods based on trapping and retaining particles in a flow and have, to our understanding, also not been reported for microfluidic enrichment applications. Inertial microfluidics is a passive separation method that has reported similar *Re*,^16,37^ but often there is a need to dilute the sample for particle focusing to occur. Instead, in this work, we use the seed particle method to capture low concentrations of microplastics for downstream analysis at higher flow rates.

#### Enrichment of Low Concentration Microplastics

**Figure 5:**
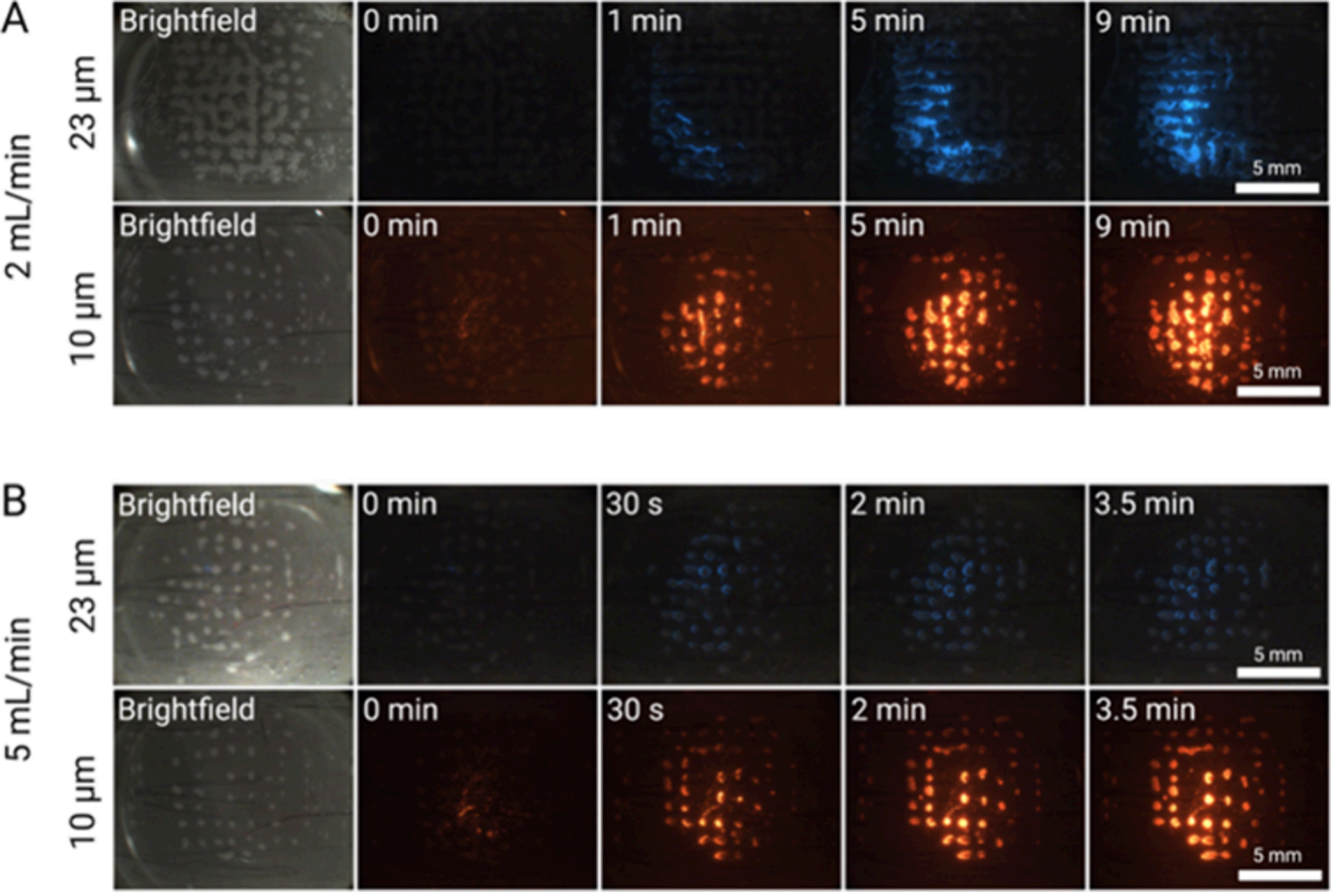
EchoGrid performance at a concentration of 10^4^ particles/mL, at the flow rates of 2 and 5 mL/min, for 23 μm (blue) and 10 μm (yellow) particles aided by silica particle seeding at a frequency of 2.020 MHz. The 23 μm particles were actuated at 11.60 V_pp_ and the 10 μm particles at 19.20 V_pp_. The bright-field images show the clusters of 10 μm silica particles. (A) Fluorescence image sequence of both particle sizes enriching at a flow rate of 2 mL/min, with the first image being the bright-field silica grid. (B) Fluorescence image sequence of both particle sizes enriching at a flow rate of 5 mL/min, with the first image being the brightfield silica grid. The fluid flows from the left (inlet) to the right (outlet).

The silica-enhanced seed particle method was tested for enrichment of low-concentration microplastics. Experimentally, we used two different sizes of particles (23 and 10 μm) to compare the capture efficiency, and due to practical limitations with the syringe pump setup, the flow rates selected were 2 and 5 mL/min. This allowed for experiments that were 10 and 4 min long using a syringe volume of 20 mL. The particle cluster enrichment is shown in Figure 5.

For both 10 and 23 μm particles, there is a clear increase in fluorescence intensity over time for both 2 mL/min (Figure 5A) and 5 mL/min (Figure 5B). From the fluorescence intensity it is apparent that the slower flow rates capture more microparticles for the given total sample volumes. It is easier to observe the difference in relative fluorescence intensity of the 23 μm particles. This is expected since particles flowing through the grid will have longer time to interact with the clusters in the field and are therefore more likely to become trapped. More importantly, we can capture and enrich the smaller 10 μm particles at concentrations that are 100-fold lower even at 25-fold higher flow rate compared to the conditions without a seed particle (see Figure 2). With the particle seed method, there is a greater surface area available within the silica particle interstitial spaces that can accommodate the particles during trapping. This increases the enrichment rate of the system for this size of particle, which was more difficult to achieve through direct capture.

**Figure 6:**
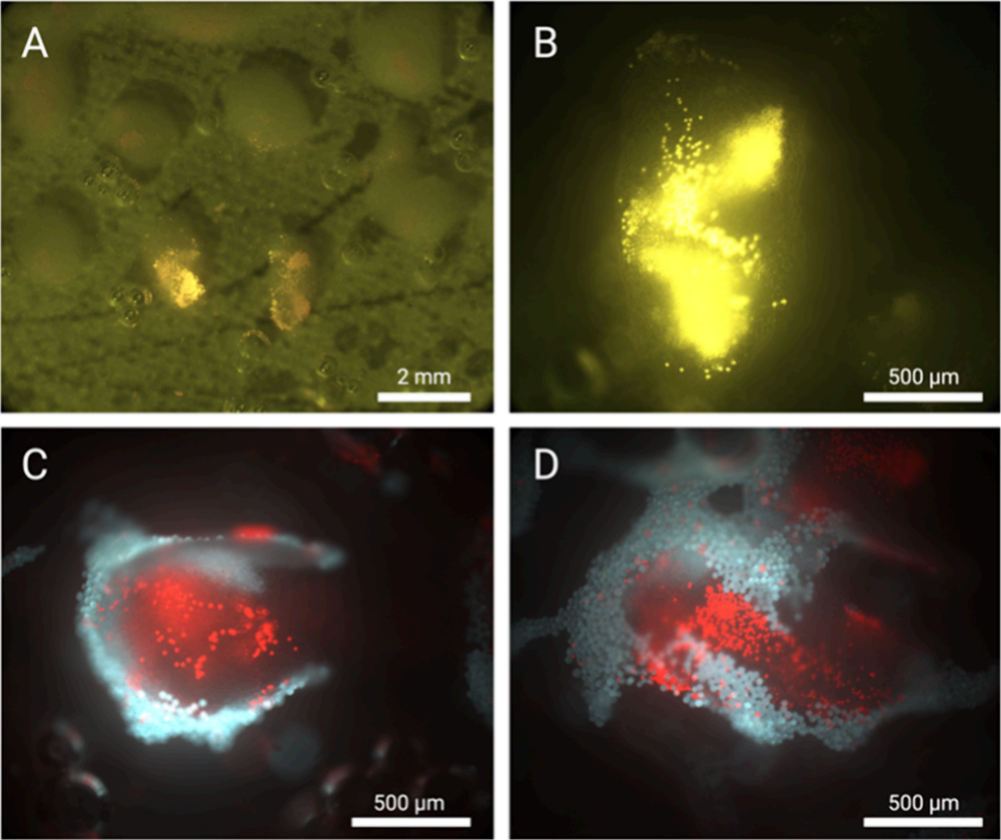
(A) Panoramic view of various clusters during enrichment, where the silica clusters can be seen as floating clouds suspended over the SDT surface. In fluorescence, the 10 μm particles (yellow) can be seen to be trapped by the silica clusters. (B) Close-up of a silica seed cluster capturing 10 μm microparticles. The yellow fluorescent microparticles can be found within the interstitial spaces of the cluster. (C and D) A different experiment where two types of particles are mixed. Examples of silica clusters that have trapped both 10 μm particles (red) and 23 μm particles (blue) are shown. These fluorescence images were taken by using a filter that can detect both particles simultaneously.

In Figure 6 we observe in greater detail how the microplastics organize themselves within and around the silica clusters, including in the case when two different particle sizes are present (Figure 6C,D). Note that the 23 μm particles are too big to enter the silica clusters’ interstitial spaces, instead encircling them and encasing them as per Figure 6C,D. The smaller 10 μm particles are able to penetrate into the silica clusters, which suggests they do not form a completely rigid barrier around the acoustic node. Instead, the silica particles are continuously finding new equilibrium positions in the cluster as they are displaced between the competing forces of the acoustic field and the flow rate.

**Figure 7:**
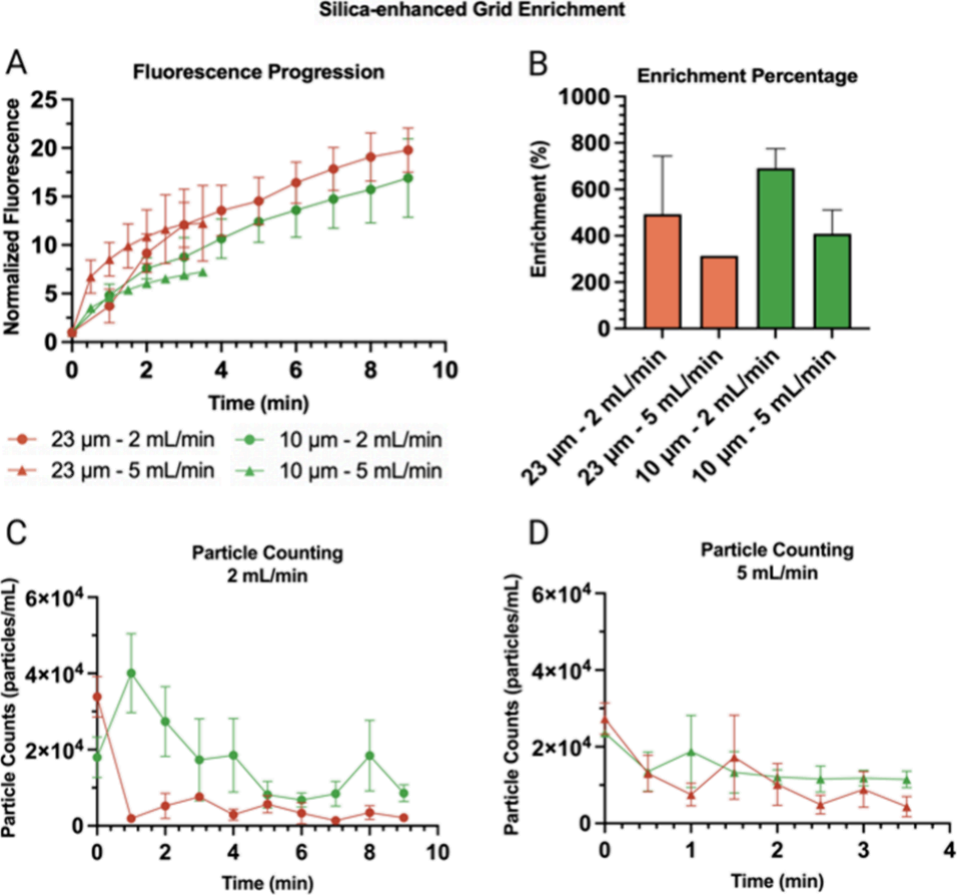
Silica-enhanced grid enrichment results. The experiments were done in triplicate. The enrichment percentages are presented in triplicate, except 23 μm–5 mL/min (one) and 10 μm–2 mL/min (two) due to difficulties in extracting the pellet with minimal volume. (A) Plot of normalized fluorescence vs time (min) for the EchoGrid performance with 23 and 10 μm microparticles at flow rates of 2 and 5 mL/min. The experiments were done in triplicate. (B) Enrichment percentage bar graph, obtained by dividing the initial inlet particle concentration by the concentration of fluorescent PS particles obtained in the clusters and extracted in a pellet. (C) Counts of 10 and 23 μm particles at the outlet were taken at discrete time points at 2 mL/min. (D) Counts of 10 and 23 μm particles at the outlet taken at discrete time points at 5 mL/min. The average values of the experiments were graphed with the standard error of the mean as error bars.

The experiments shown in Figure 5 are quantified in Figure 7. Here, we observe that there is an improvement in terms of capture performance compared with the seedless version. The fluorescence increase recorded from the images at each time point can be seen in Figure 7A. The enrichment percentage of the pellet across experimental time can be seen in Figure 7B, and the particle counting section for each flow rate can be found in panels C and D. Specifically, in the case of the 5 mL/min experiments, both particle sizes enrich more rapidly at first but then at a lower rate as the experiment progresses (Figure 7A). This result suggests the existence of an optimal combination of flow rate, silica grid density, and particle concentration in which one can maximize the enrichment speed and capacity of the system.

The enrichment percentage (Figure 7B) is obtained by dividing the initial solution’s particle count by the particle count of the clusters extracted from the device in a pellet. Here, we observe a greater absolute enrichment at 2 mL/min for both particle sizes, which agrees with the progression of normalized fluorescence in terms of the number of particles captured by the grid. The maximum enrichment reached by these experiments was 770% in the case of 23 μm–2 mL/min. It should be noted that this requires accurate extraction of the pellet, which may sometimes carry excess buffer, reducing the final enrichment percentage at best or mismanaging the sample at worst. In terms of particle counting, the 23 μm particles at 2 mL/min (Figure 7C) show a capture efficiency of 85.8% at 9 min. For the 10 μm particles at 2 mL/min, the process reaches 52.3% capture efficiency at 9 min. For the 5 mL/min experiments (Figure 7D), in terms of 23 and 10 μm particles, the obtained capture percentages are 84.1% and 51.4%, respectively, by the end of the experiment. The relative value of these results is consistent with what was obtained in the initial counting experiments of this paper, except now these are much higher flow rates (25× higher, from 200 μL/min to 5 mL/min) and lower concentrations (100× lower, from 10^6^ particles/mL to 10^4^ particles/mL).

Another relevant measure is the enrichment percentage of the pellet per unit of time. It expresses how much the concentration of captured microplastics increases in the central cluster pellets every minute. On average, for 23 μm, we obtain a pellet enrichment of 49.3%/min at 2 mL/min and 76.8%/min at 5 mL/min. For the 10 μm particles, we obtain a pellet enrichment of 89.6%/min at 2 mL/min and 116.7%/min at 5 mL/min. We observe that the higher flow rate (5 mL/min) enriches more quickly but captures particles less efficiently. The concentration of the pellet is doubled every 78 s for 23 μm particles and every 51 s for 10 μm particles for the higher flow rate at 5 mL/min.

This enrichment performance is highly relevant for microplastic monitoring toward environmental applications, showing increased throughput and efficiency when compared to other acoustic^24,25,38^ and even inertial^39^ devices. It does so at the relatively low concentration of 10^4^ particles/mL, as has been found in bottled water.^40^ Based on the high concentration silica solution (10^8^ particles/mL) used to fill the channel before the creation of the preseeded grid and the volume of the channel (309 mm^3^), we estimate that the system has the capacity for a minimum of 3.09 × 10^7^ of these particles. Considering the difference in magnitude between this value and the real-life concentration of 10^4^ particles/mL (3.09 × 10^3^ inside the channel volume), we conservatively estimate that at least 100 L of sample would have to be processed before the capacity of the chip is exhausted. This number will vary according to size distribution of the sample and also on the size of the preseeded clusters, which can be tuned according to need.

Although real water samples cannot be detected using fluorescence, as done in this work, the EchoGrid was designed to be compatible with other detection methods. The combination of this module with analytical techniques such as Raman spectroscopy will be attractive for an integrated fit-for-purpose microplastic monitoring platform.

## Conclusions

In this article, a novel acoustofluidic device for environmental applications involving microplastics is presented. This device, termed the EchoGrid, uses low-cost materials such as PDMS and glass integrated with a surface displacement transducer (SDT) to create a platform that surpasses traditional microfluidics in terms of microparticle enrichment at high throughput. The reusable device was capable of directly capturing 2, 10, and 23 μm particles at high concentrations in a stable, spatially well-defined grid structure that is reproducible and tunable across experiments. To address the challenge of low concentration samples at a high flow rate, the silica-enhanced seed particle method was developed. This is a novel approach, as the seed particle method is typically used with submicrometer particles. The extremely high flow rates in microfluidics (50 mL/min) at which the silica grid can be retained in the channel are, to the best of our knowledge, unprecedented. Using this silica-enhanced seed particle method for microparticles, it is possible to enrich 23 and 10 μm particles at low concentrations (10^4^ particles/mL), even at the considerable flow rates of 2 and 5 mL/min, while keeping a spatially well-defined grid. These flow rates allow the processing of a 20 mL syringe in a mere 10 and 4 min, respectively. In conclusion, the EchoGrid overcomes unsolved technological barriers in acoustofluidics toward an environmental microplastic monitoring solution.

## Supporting Information Available

**Figures S1–S4**: methodological details and figures detailing controls and extra experiments; **Table S1**: the velocity and Reynolds Number values relating to the flow rates used during the work (PDF)
