# Supplementary material for: EchoGrid: High-Throughput Acoustic Trapping for Enrichment of Environmental Microplastics
Source: Anal Chem. 2024 May 25;96(23):9493–502. doi: 10.1021/acs.analchem.4c00933 (PMC11170556; doi:10.1021/acs.analchem.4c00933)
Supplement: Supplementary file 1 — ac4c00933_si_001.pdf [file ac4c00933_si_001.pdf]

Supporting Information

EchoGrid: High-Throughput Acoustic Trapping for Enrichment of Environmental Microplastics

Martim Costa[1], Björn Hammarström[2], Liselotte van der Geer[1], Selim Tanriverdi[1], Haakan N. Joensson[1], Martin Wiklund[2] and Aman Russom*[1,3]

1 KTH Royal Institute of Technology, Division of Nanobiotechnology, Department of Protein Science, Science for Life Laboratory, Solna, Sweden

2 KTH Royal Institute of Technology, Department of Applied Physics, Science for Life Laboratory, Solna, Sweden

3 AIMES – Center for the Advancement of Integrated Medical and Engineering Sciences at Karolinska Institutet and KTH Royal Institute of Technology, Stockholm, Sweden

***Corresponding Author: Aman Russom (aman@kth.se)**

Table of Contents:

Figure S1: Figure detailing the relevant acoustic forces present within a cluster, as well as the methodological details for experiments using direct capture of microplastics and the silica-enhanced seed particle method.

Figure S2: Figure depicting the different modes of operation of the acoustic field in the EchoGrid with 2 µm particles.

Figure S3: Figure with the fluorescence images from the direct capture experiments at 10^4 particles/mL.

Figure S4: Figure containing the particle counting graphs of the experiments at 10^6 particles/mL and 10^4 particles/mL.

Table S1: Table of the calculated values of the Reynolds numbers and fluid velocities (mm/s).

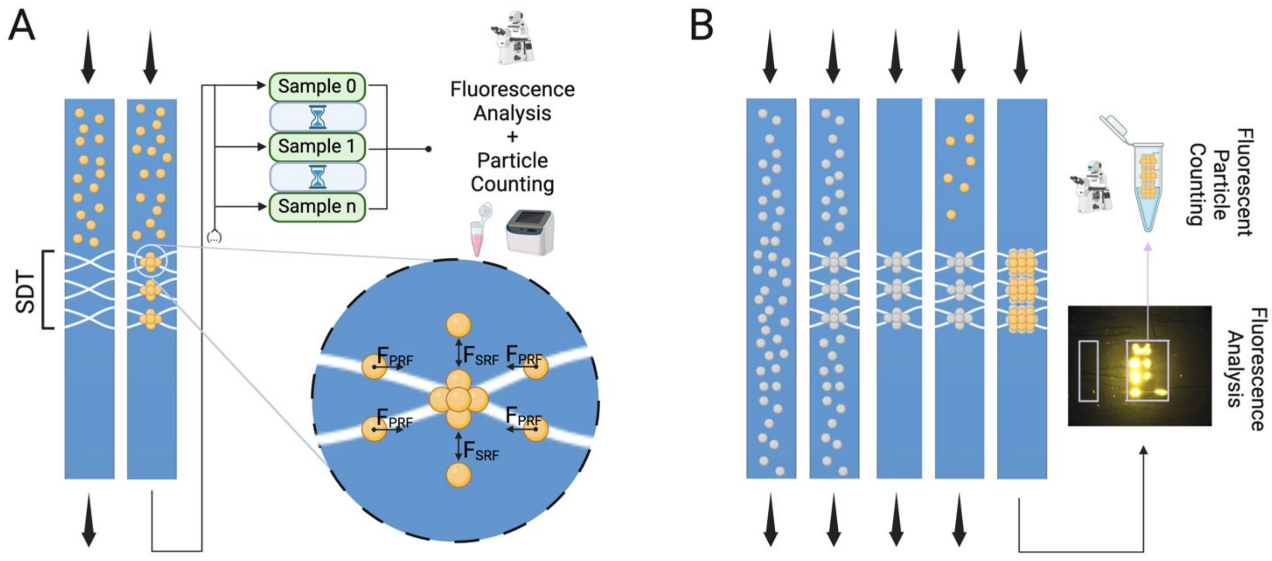

Figure S1. (A) Direct capture of microplastics. Microparticles were captured and enriched by acoustic trapping above the SDT. Fluid samples and fluorescent images were collected with regular intervals for analysis. The inset shows how particles are directed to and retained in the trapping site by the primary acoustic radiation forces (PRF), and how secondary acoustic radiation forces (SRF) contribute by attracting particles to each other. **(B)** Silica-enhanced seed particle method capture of microplastics was done by preloading the acoustic trap with silica particles. Excess silica was washed out at high flow rate (15 mL/min) to retain silica only in the trapping hotspots. Microplastics were subsequently captured in and around the silica particles and the enrichment was quantified in a similar fashion.

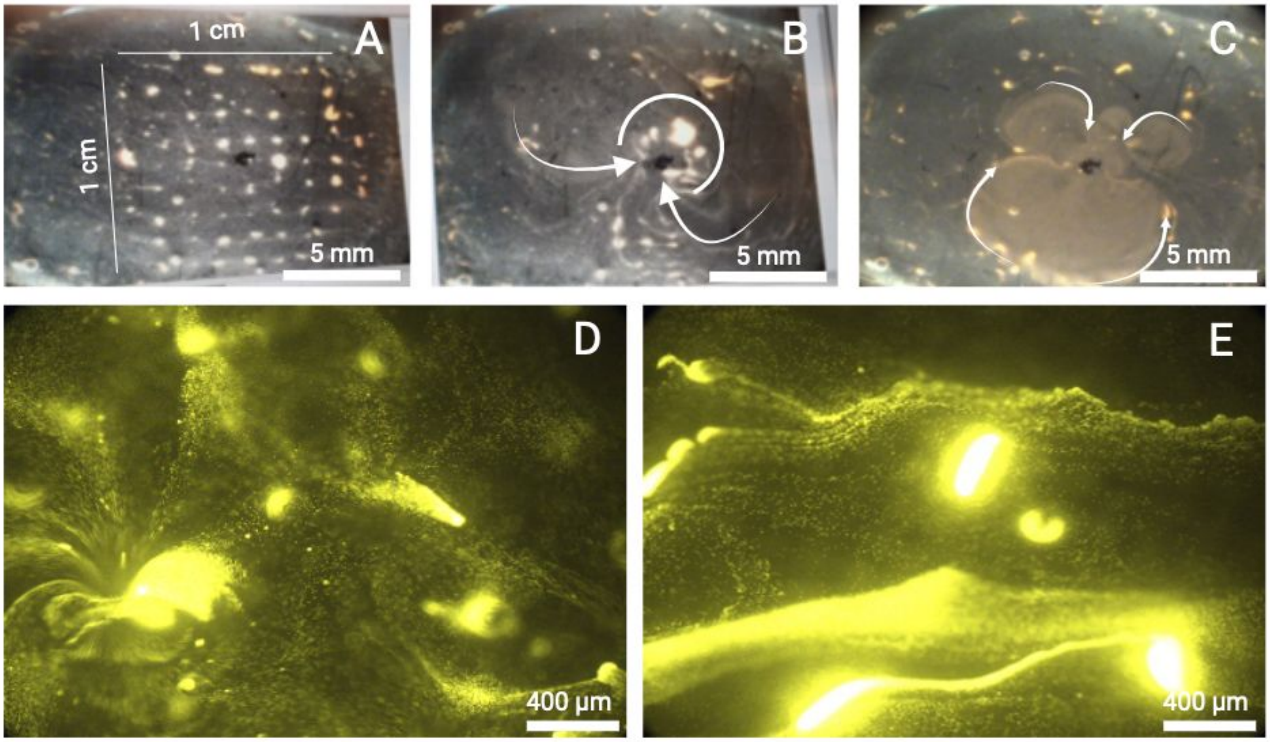

Figure S2. Operation of the device during pulling flow at 20 µL/min, using 2 µm particles (yellow) **(A)** Grid formation over the transducer, f: 1.767 MHz at 20 µL/min **(B)** Larger central cluster formation with visible streaming lines, f: 1.760 MHz at 20 µL/min **(C)** Acoustic streaming (white arrows) during flow, f: 1.751 MHz at 20 µL/min. **(D)** Close-up of acoustic streaming flowlines **(E)** Close-up of several 2 µm particle clusters.

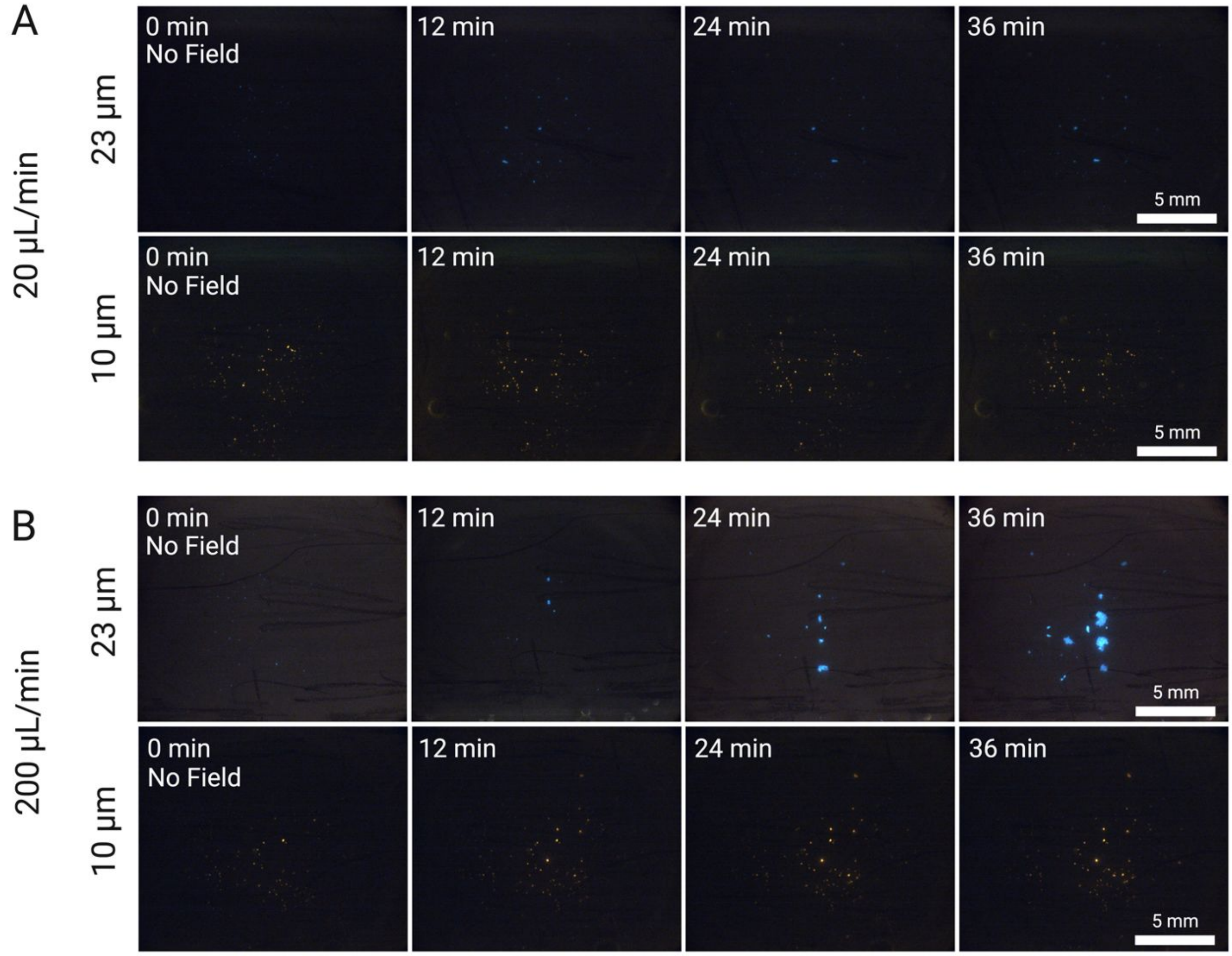

Figure S3. The EchoGrid performance at a concentration of 10^4 particles/mL. **(A)** Fluorescence image sequence for the 23 (blue) and 10 (yellow) µm particles at 20 µL/min. **(B)** Fluorescence image sequence for the 23 and 10 µm particles at 200 µL/min. Actuation parameters: 11.60 V_{pp} for 23 µm particles and 19.20 V_{pp} for 10 µm particles, both at a frequency of 2.020 MHz. The fluid flows from left (inlet) to right (outlet). A single experiment and control pair at 200 µL/min for 10 µm was done to observe whether any capture was observable, which was not the case. The remaining experiments were done in triplicates.

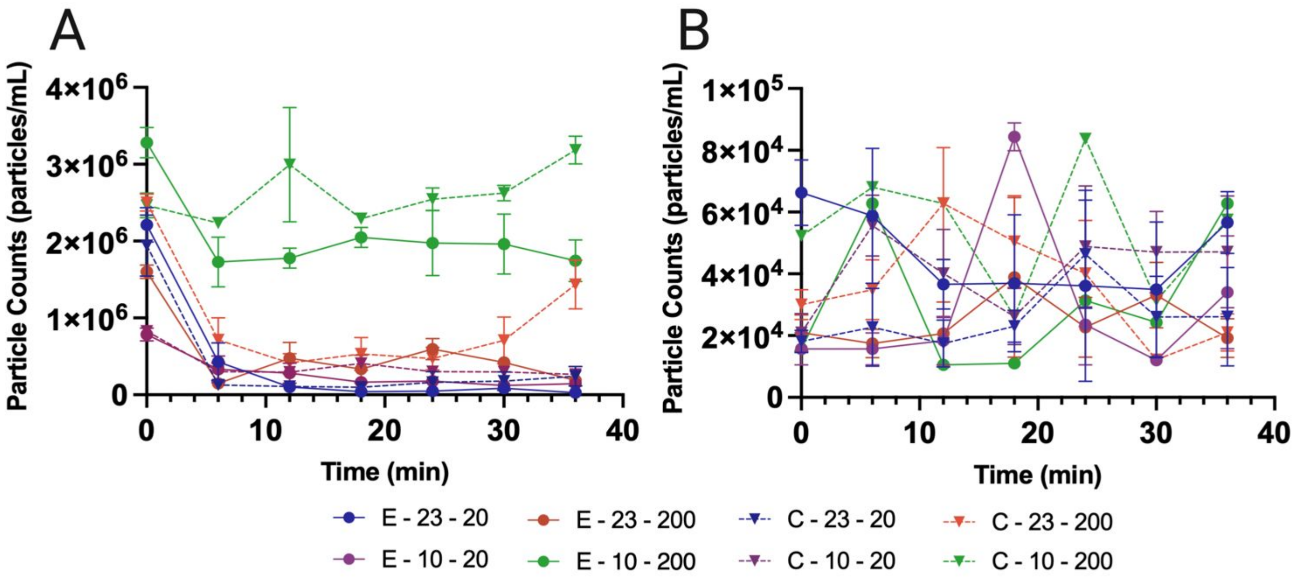

Figure S4. (A) Particle counting from the outlet at a concentration of 10^6 particles/mL, for both flow rates and particle sizes. **(B)** Particle counting from the outlet at a concentration of 10^4 particles/mL, for both flow rates and particle sizes. All experiments were done in triplicates. except for the E – 10 – 200 and C – 10 – 200 trials at 10^4 particles/mL concentration.

Flow rate (mL/min)	2	5	10	20	35	50
Reynolds Number	4.1	10.1	20.3	40.6	60.8	101.4
Velocity (mm/s)	10.7	26.6	53.3	106.5	186.4	266.2

Table S1. Calculated Reynolds Numbers for the system at the center of the trapping grid, as well as fluid velocity.
